# High Blood Pressure Prevalence, Awareness, Control, and Associated Factors in a Low-Resource African Setting

**DOI:** 10.3389/fcvm.2019.00119

**Published:** 2019-08-30

**Authors:** Olivier Pancha Mbouemboue, Tsougmo Jacques Olivier Ngoufack

**Affiliations:** ^1^Department of Biomedical Sciences, Faculty of Science, University of Ngaoundere, Ngaoundere, Cameroon; ^2^General Medicine Service, Ngaoundere Regional Hospital, Ngaoundere, Cameroon; ^3^Hypertension and Diabetes Unit, Ngaoundere Regional Hospital, Ngaoundere, Cameroon

**Keywords:** Ngaoundere, Cameroon, hypertension, prevalence, awareness, treatment, control

## Abstract

**Background and Objectives:** Recent and contextualized data are needed to improve hypertension management known as a major cardiovascular disease risk factor regardless of the geographical area. This study aimed at assessing the prevalence of hypertension, awareness of hypertensive status, treatment, and control of hypertension as well as assessing the factors associated with risk of hypertension and awareness of hypertensive status in the population of Ngaoundere.

**Methods:** This was a community based cross sectional study carried out from February to December 2016. A three-stage sampling method was used for recruitment of participants. Demographic, clinical, and biological data were collected and analyzed using Statistical Package for Social Sciences version 20.0. Statistical significance was set at *P* < 0.05.

**Results:** In total, 948 participants were included in the study. The overall prevalence of hypertension was 46.94% (*n* = 445). Fraction of hypertensive participants who were aware of their status was 36.85% (*n* = 164). Among them, 39 (23.78%) were getting treatment and the control rate of treated hypertensives was 30.56%. Age, marital status, family history of hypertension, overweight, and high serum triglyceride level were identified as independent predicting factors of hypertension, whereas female gender, age, personal history of stroke or diabetes, family history of hypertension or heart failure, overweight, and abdominal obesity were those of hypertension awareness.

**Conclusion:** The present study revealed high prevalence, extremely low awareness, treatment, and control rates of hypertension in Ngaoundere community setting.

## Introduction

Hypertension is a major cardiovascular disease risk factor and its prevalence has shown a rapid increase in Sub-Saharan Africa (1–6). This prevalence can be substantially reduced through prevention, early diagnosis, and proper management. According to World Health Organization (WHO), recent and contextualized epidemiological data are needed for this purpose. However, cardiovascular disease estimates remain uncertain in low-income countries because of the scarcity of epidemiological studies in some areas (5–7). A previous study showed that hypertension is the most frequent encountered cardiovascular condition and the main cause of major cardiovascular events in Ngaoundere hospital setting (8). It is then possible to hypothesize that an important cardiovascular risk factor such as hypertension still yet not well managed, and that cardiovascular disease prevention remain a great public health challenge in this region. Clearly, lack of evidence data on cardiovascular disease risk factors worsens the situation, thus the need to improve availability of reliable epidemiologic data on cardiovascular disease in this area. This study aimed at assessing the prevalence of hypertension, awareness of hypertensive status, treatment, and control of hypertension as well as assessing the factors associated with risk of hypertension and awareness of hypertensive status in population of Ngaoundere in Cameroon.

## Methods

### Study Population and Selection Criteria

A community based cross sectional study was conducted from February to December 2016 in Ngaoundere, Cameroon. Ngaoundere is the chief town of Adamawa Region. It is located in the high altitude guinea savannah ecological zone and is characterized by one rainy season from April to October and one dry season from November to March. The Foulbes, the Haoussas, the Gbaya, the Tikar, the Mboum, and the Dii are the leading ethnic groups in the area. The main economic activities are dairy farming and agriculture. Participants were selected following a three-stage cluster sampling method, among people aged 20 years or above and who have been living in the study area for at least 1 year. Individuals on corticosteroid therapy, hormonal contraceptives, or presenting with chronic kidney disease were excluded. Details of sampling procedure are described in a previous work (9).

### Study Variables

Demographic (sex, age, marital status), anthropometric (body mass index, waist circumference), clinical (medical history, blood pressure, awareness, treatment, and control of hypertension), and biological variables (fasting serum glucose, triglyceride, and total cholesterol levels) were studied. Overweight and obesity were defined as body mass index ranges 25–29.9 and >29.9 kg/m^2^, respectively (10). Abdominal obesity was defined as waist circumference >102 cm for men and 88 cm for women (10, 11). Hypertension was defined as blood pressure >140/90 mmHg at the time of study or antihypertensive therapy for at least 15 days (12, 13). All participants who answered yes to the question “are you suffering from hypertension?” Were considered as aware of hypertension. Hypertension was considered to be controlled in all treated hypertensive subjects who had at the time of the study a blood pressure <140/90 mmHg.

Diabetes was defined as fasting blood glucose >1.26 g/L (14), or a self-report of previous diagnosis of diabetes. National Cholesterol Education Program Expert Panel on Detection, Evaluation, and Treatment of High Blood Cholesterol in Adults, Adult Treatment Panel III Criteria were used to define high serum triglyceride and total cholesterol levels (more than 200 mg/dL for triglycerides; more than 240 mg/dL for total cholesterol) (15).

### Data Collection

Demographic data and medical history were collected using a semi-structured questionnaire. Following The Seventh Report of the Joint National Committee on Prevention, Detection, Evaluation, and Treatment of High Blood Pressure recommendations, blood pressure was measured in participants on sitting position after at least 5 min of rest (12). The investigators also ensured that the participants had not consumed tobacco or caffeinated beverages, had not practiced physical activity for at least 30 min prior to blood pressure measurement (12). Blood pressure was measured using an electronic blood pressure monitor type OMROM® HEM-8712. Two measurements were made with a time lapse of 3–5 min and the mean of the two measures was used for analysis. Weight, height, and waist circumference measurements were done following the WHO STEPwise approach (10). A TIAN SHAN®-2003B brand electronic weigh scale, a local stadiometer, and a non-stretchable flexible graduated tape were, respectively, used for this purpose. Body mass index was calculated by dividing weight (in kilograms) by the square of height (in meters) (16). Blood samples were collected in each participant after 8–12 h overnight fast. From those samples, fasting blood glucose, triglyceride, and total cholesterol levels were estimated via colorimetric reactions and spectrophotometric readout (9).

### Data Analysis

Data were analyzed using Statistical Package for Social Sciences version 20.0. Pearson's Chi-square and Fisher's exact tests were used to compare proportions. Univariate and multivariate logistic analyses were used to assess the crude and adjusted effect of seemingly significant predictors of hypertension and awareness of hypertension. A *P* < 0.05 was considered as statistically significant.

### Ethical Considerations

The study was approved by the Ethical Committee of the University of Ngaoundere, the Local Ethical Committee (Reference no. 1121/L/RC/RA/DSP/HR/NGD/CLE) and the National Ethics Committee for Human Health Research. It was also approved by the Regional Delegate of Public Health for Adamawa Region (Reference no. 651/l/RA/DSP/SAG/BPF/NGD). Prior to individual data collection, each participant was clearly informed about the study and freely signed a written consent form.

## Results

A total of 948 participants were included in the study, comprising 464 (48.95%) men and 484 (51.05%) women. Their mean age was 38.82 ± 14.92 years (range 20–87). Most of the participants were married (Table 1).

**Table 1:** Baseline characteristics of the study participants, hypertension prevalence, and awareness.

**Variables**	**Variable distribution** ***n* (%)**	**Hypertension prevalence** ***n* (%)**	**Hypertension awareness**	
			**Among hypertensives** ***n* (%)**	**Among all the participants** ***n* (%)**
Overall	948 (100)	445 (46.94)	164 (36.85)	164 (17.30)
Gender				
Male	464 (48.95%)	209 (45.04)	59 (28.23)	59 (12.72)
Female	484 (51.05%)	236 (48.76)	105 (44.49)	105 (21.69)
Age				
20–39 years	572 (60.34)	189 (33.04)	51 (26.98)	51 (9.96)
40–64 years	301 (31.80)	196 (65.12)	77 (39.29)	77 (25.58)
≥65 years	75 (7.91)	60 (80.00)	36 (60.00)	36 (48.00)
Marital status				
Singles	276 (29.11)	75 (27.17)	19 (25.33)	19 (6.88)
Divorced	51 (5.38)	26 (5098)	12 (46.15)	12 (23.53)
Married	585 (61.71)	314 (53.98)	118 (37.58)	118 (20.21)
Widows	36 (3.80)	30 (81.08)	15 (50.00)	15 (40.54)
Personal history of				
Stroke	16 (1.69)	13 (81.25)	9 (69.23)	9 (56.25)
CAD	8 (0.84)	3 (37.50)	2 (66.67)	2 (25.00)
Family history of				
Hypertension	318 (33.54)	170 (53.46)	79 (46.47)	79 (24.84)
Stroke	56 (5.90)	31 (55.36)	16 (28.57)	16 (28.51)
CAD	40 (4.21)	20 (50.00)	9 (22.50)	9 (22.50)
Body mass index				
Normal	523 (55.17)	237 (41.36)	78 (32.91)	78 (14.91)
Overweight	239 (25.21)	140 (54.69)	62 (44.28)	62 (25.94)
Obesity	111 (11.71)	68 (57.14)	24 (35.29)	24 (21.62)
Abdominal obesity				
No	749 (79.01)	323 (45.74)	108 (33.43)	108 (14.42)
Yes	199 (20.99)	122 (61.31)	56 (45.90)	56 (28.14)
Diabetes				
No	895 (94.41)	427 (46.56)	156 (36.53)	156 (17.43)
Yes	53 (5.59)	11 (73.33)	6 (54.55)	6 (11.32)
Serum triglyceride				
<1.5 g/L	823 (86.81)	415 (48.11)	151 (36.38)	151 (18.34)
>1.5 g/L	125 (13.19)	35 (71.43)	13 (37.14)	13 (10.40)
Serum total cholesterol				
<2 g/L	500 (52.74)	308 (43.07)	114 (37.01)	114 (22.28)
≥2 g/L	448 (47.26)	137 (56.61)	50 (36.49)	50 (11.16)

*CAD, coronary artery disease*.

### Hypertension Prevalence and Awareness

The overall prevalence of hypertension was 46.94% (*n* = 445) in the study population, 45.04% in men and 48.76% in women. Older participants and those with high body mass index had higher prevalence of hypertension than younger participants and those with lower body mass index. Diabetic participants and those having a personal history of stroke, a family history of hypertension, abdominal obesity, high serum triglyceride, and total cholesterol levels recorded higher prevalence of hypertension than their counterparts (Table 1).

A proportion of 36.85% of hypertensive participants (17.30% of whole study population) were aware of their status. Women were more aware of their hypertensive status than men (44.49 vs. 28.23%). Moreover, older participants were more aware of their status than youngers (Table 1).

### Hypertension Treatment and Control

A total of 39 (23.78%) aware hypertensive participants were on blood pressure lowering medication. So, only 8.76% of all the hypertensive participants were treated. The use of drug treatment for hypertension was higher among women than men (30.48 vs. 11.86%). It was also higher in older participants than youngers. Similarly, it was higher in hypertensive participants with a personal or family history of coronary artery disease and in those with abdominal obesity than those without (Table 2).

**Table 2:** Hypertension treatment and control rates in the study population.

**Variables**	**Treated hypertension**		**Controlled hypertension**	
	**Among aware hypertensives** ***n* (%)**	**Among all hypertensives** ***n* (%)**	**Among treated hypertensives** ***n* (%)**	**Among all hypertensives** ***n* (%)**
Overall	39 (23.78)	39 (8.76)	11 (30.56)	11 (0.02)
Gender				
Male	7 (11.86)	7 (3.35)	2 (28.57)	2 (0.96)
Female	32 (30.48)	32 (13.56)	9 (31.03)	9 (3.81)
Age				
20–39 years	9 (17.65)	9 (4.76)	4 (44.44)	4 (2.12)
40–64 years	19 (24.68)	19 (9.69)	6 (31.57)	6 (3.06)
≥65 years	11 (30.56)	11 (18.33)	1 (9.09)	1 (1.67)
Personal history of *Stroke*				
No	38 (24.52)	38 (8.80)	10 (26.31)	10 (2.31)
Yes	1 (11.11)	1 (7.69)	1 (100.00)	1 (7.69)
*CAD*				
No	38 (23.46)	38 (8.59)	11 (28.95)	11 (2.48)
Yes	1 (50.50)	1 (33.33)	0 (0.00)	0 (0.00)
*Hypertension*				
No	18 (21.18)	18 (6.54)	6 (33.33)	6 (4.72)
Yes	21 (26.58)	21 (12.35)	5 (23.81)	5 (2.94)
Family history of *Stroke*				
No	35 (23.65)	35 (8.45)	9 (25.71)	9 (2.31)
Yes	4 (25.00)	4 (12.90)	2 (50.00)	2 (6.45)
*CAD*				
No	37 (28.87)	37 (8.70)	11 (29.72)	11 (2.58)
Yes	2 (22.22)	2 (10.00)	0 (0.00)	0 (0.00)
Body mass index				
Normal	15 (19.23)	15 (6.75)	5 (31.25)	5 (0.02)
Overweight	18 (29.03)	18 (12.85)	3 (16.67)	3 (2.14)
Obesity	6 (25.00)	6 (8.82)	3 (50.00)	3 (4.41)
Abdominal obesity				
No	20 (18.52)	20 (6.19)	6 (30.29)	6 (1.88)
Yes	19 (33.93)	19 (15.57)	5 (26.23)	5 (4.10)
Diabetes				
No	34 (25.00)	34 (8.42)	10 (28.57)	10 (2.34)
Yes	5 (17.86)	5 (12.20)	1 (20.00)	1 (9.09)
Serum triglyceride				
<1.5 g/L	31 (23.13)	31 (7.95)	9 (28.13)	9 (2.41)
>1.5 g/L	8 (36.36)	8 (8.57)	2 (33.33)	2 (2.86)
Serum total cholesterol				
<2 g/L	14 (20.29)	14 (6.82)	6 (42.86)	6 (2.92)
≥2 g/L	25 (35.71)	25 (10.95)	5 (20.00)	5 (1.46)

*CAD, coronary artery disease*.

Thiazide-type diuretics and angiotensin-converting enzyme inhibitors were the most frequent antihypertensive agent used for hypertension treatment in the study population. These drug classes were used by 41.03% (*n* = 16) and 33.33% (*n* = 13) of the treated hypertensive participants, respectively. The least used antihypertensive agent was angiotensin receptor blockers (Table 3).

**Table 3:** Antihypertensive drugs and therapy type used by treated participants.

**Parameters**	***n***	**%**
Antihypertensive agents		
Thiazide-type diuretics	16	41.03
Angiotensin-converting enzyme inhibitor	13	33.33
Calcium channel blockers	6	15.38
Centrally acting antihypertensive agents	6	15.38
Beta-blocking agents	5	12.82
Other diuretics	4	10.26
Angiotensin receptor blockers	2	5.13
Therapy type		
Monotherapy	24	61.54
Bi-therapy	15	38.46

The hypertension control rate was 30.56% (*n* = 11) among treated hypertensives and 0.02% among the overall hypertensive participants. The level of blood pressure control was higher in women (31.03%) than in men (28.57%). It was also higher in participants with personal or family history of stroke than those without (Table 2).

### Factors Associated With Hypertension

The association of independent variables with hypertension was investigated using both univariate and multivariate logistic regression techniques. In univariate logistic regression analysis; age, gender, marital status, personal history of stroke or diabetes, family history of hypertension, body mass index, waist circumference, diabetes, serum triglyceride, and total cholesterol levels showed significant associations with hypertension (Table 4). Therefore, all those variables were used in multivariate analysis, which showed that age, marital status and family history of hypertension, body mass index, and serum triglyceride level were independently associated with the odds of having hypertension. Participants aged ≥ 65 years and those aged 40–64 years were five times (AOR = 5.52; *P* < 0.01) and two times (AOR = 2.65; *P* < 0.01), respectively, more likely to present hypertension than those aged 20–39 years (Table 4).

**Table 4:** Factors associated with hypertension.

**Variables**	**COR (95% CI)**	***P*-value**	**AOR (95% CI)**	***P*-value**
Age (years)				
20–39	1		1	
40–64	3.78 (2.82–5.08)	<0.01^*^	2.65 (1.88–3.73)	<0.01^*^
65 and above	8.11 (4.48–14.65)	<0.01^*^	5.52 (2.87–10.60)	<0.01^*^
Marital status				
Singles	1		1	
Divorced	2.79 (1.51–5.13)	<0.01^*^	1.38 (0.70–2.74)	0.36
Married	3.12 (2.28–4.25)	<0.01^*^	1.69 (1.18–2.42)	<0.01^*^
Widows	11.49 (4.84–27.26)	<0.01^*^	3.60 (1.40–9.26)	0.01^*^
Personal history of stroke				
No	1		1	
Yes	5.02 (1.4–17.72)	0.01^*^	3.53 (0.90–13.85)	0.07
Personal history of diabetes				
No	1		1	
Yes	4.83 (2.30–10.15)	<0.01^*^	2.36 (0.43–12.98)	0.33
Family history of hypertension				
No	1		1	
Yes	1.48 (1.13–1.94)	<0.01^*^	2.13 (1.54–2.96)	<0.01^*^
Body mass index				
Normal	1		1	
Overweight	1.71 (1.27–2.30)	<0.01^*^	1.52 (1.06–2.17)	0.02^*^
Obesity	1.89 (1.27–2.82)	<0.01^*^	1.31 (0.74–2.32)	0.35
Abdominal obesity				
No	1		1	
Yes	2.09 (1.52–2.88)	<0.01^*^	1.14 (0.70–1.85)	0.59
Diabetes				
No	1		1	
Yes	4.15 (2.15–8.01)	<0.01^*^	1.55 (0.34–7.11)	0.57
Serum triglycerides				
Normal	1		1	
High	2.79 (1.82–4.29)	<0.01^*^	2.24 (1.13–4.46)	0.02^*^
Serum total cholesterol				
Normal	1		1	
High	1.58 (1.22–2.05)	<0.01^*^	1.06 (0.76–1.50)	0.72

*COR, crude odds ratio; AOR, adjusted odds ratio; 95% CI, 95% confidence interval*.

* *P <0.05*.

### Factors Associated With Hypertension Awareness

In univariate logistic regression analysis, female gender, age, personal history of stroke or diabetes, family history of hypertension or heart failure, body mass index, and abdominal obesity showed significant associations with hypertension awareness (Table 5). Those factors were then used in multivariate logistic regression analysis, which revealed that female gender, age, personal history of diabetes, and family history of hypertension were independently associated with hypertension awareness. Thus, female participants were more likely to be aware of their hypertensive status than males (AOR = 0.49; *P* = 0.02). Moreover, Participants aged ≥ 65 years were four times (AOR = 4.24; *P* < 0.01) more likely to be aware of their hypertensive status than those aged 20–39 years (Table 5).

**Table 5:** Factors associates with hypertension awareness.

**Variables**	**COR (95% CI)**	***P-value***	**AOR (95% CI)**	***P-value***
Gender				
Female	1		1	
Male	0.49 (0.33–0.73)	<0.01^*^	0.57 (0.35–0.92)	0.02^*^
Age (Years)				
20–39	1		1	
40–64	1.75 (1.14–2.69)	0.01^*^	1.67 (1.04–2.66)	0.03^*^
65 and above	4.06 (2.21–7.45)	<0.01^*^	4.24 (2.16–8.32)	<0.01^*^
Personal history of stroke				
No	1		1	
Yes	4.02 (1.22–13.27)	0.02^*^	3.57 (0.98–13.02)	0.05
Personal history of diabetes				
No	1		1	
Yes	5,96 (2.72–13.02)	<0.01^*^	5.40 (2.32–12.55)	<0.01^*^
Family history of hypertension				
No	1		1	
Yes	1.94 (1.31–2.88)	<0.01^*^	2.18 (1.39–3.41)	<0.01^*^
Family history of heart failure				
No	1		1	
Yes	1.68 (1.06–2.68)	0.03^*^	1.45 (0.87–2.43)	0.15
Body mass index				
Normal	1		1	
Overweight	1.62 (1.05–2.49)	0.03^*^	1.44 (0.88–2.39)	0.15
Obesity	1.11 (0.63–1.96)	0.71	0.71 (0.33–1.52)	0.38
Abdominal obesity				
No	1		1	
Yes	1.69 (1.10–2.58)	0.02^*^	1.12 (0.60–2.08)	0.73

*COR, crude odds ratio; AOR, adjusted odds ratio; 95% CI, 95% confidence interval*.

* *P <0.05*.

## Discussion

The main findings of this study are the higher prevalence of hypertension and its significant association with age and marital status, the lower hypertension awareness, treatment, and control in Ngaoundere urban community. These findings highlight the importance of reliable data in the hypertension prevention and control process in our setting. Bearing in mind the higher but not currently apparent prevalence of hypertension, it is necessary to consider hypertension as a huge public health problem in this region.

### Hypertension Prevalence and Associated Factors

The prevalence of hypertension is gradually increasing in sub-Saharan Africa (17, 18). In this region, the prevalence of hypertension increased from 19.7 to 30.8% between 1990 and 2010 (19). Recent studies indicate a prevalence of hypertension among adult subjects close to 45% in African urban areas. Hypertension prevalence rates of 38.9, 45.2, 47, and 41.4%, have been reported in similar study populations in South Africa (20), Angola (21), Nigeria (22), and Democratic Republic of Congo (23), respectively. The 46.94% prevalence rate of hypertension found in the study is close to those reported in many other African countries, but higher as compared to those reported in higher-income countries for similar age group populations (2, 24), suggesting an influence of income level in the prevention and treatment of cardiovascular diseases. In Israel for example, the well-developed health system including national health insurance coverage have substantially increased access to treatment for the low income groups and so, have improved the prevention and control of hypertension (24).

Results of the study indicated that age, marital status and family history of hypertension, body mass index, and serum triglyceride level were factors independently associated with the odds of having hypertension.

Age has been identified as a determinant of blood pressure level (3, 6, 20, 24–26). In the present study, the prevalence of hypertension was 33.04% in participants aged 20–39 years, 65.12% in subjects aged 40–64 years and 80.00% in subjects aged 65 years and above. Moreover, multivariate analysis showed that participants aged ≥ 65 years and those aged 40–64 years were more likely to have hypertension than those aged 20–39 years. These results are consistent with those of previous studies and confirm the relationship between age and hypertension (24–26).

There was also a significant association between hypertension and marital status. Multivariate analysis showed that married and widowed participants were more likely to be hypertensive than single participants. This association could be explained by the influence of age, assuming that widows are commonly older than marrieds, whom are commonly older than singles. Marital status has previously shown association with hypertension (24, 27, 28), being married affecting lifestyle, health-related behaviors, psychosocial support and adherence to dietary advice, and medical treatment (24, 29). However, level and direction of the effect differ from a study to another, being sometimes conflicting. Marriage failure have been said to inflict a great psychological stress, which is involved in increasing the risk of having hypertension (27). In a multicenter study carried out in six adjacent Middle Eastern Gulf countries, Hadi Khafaji et al. found that widowed marital status was associated with a higher cardiovascular risk profile (29). Contrariwise, marriage have been described by other authors as factor mitigating or exacerbating exposure to cardiovascular risk factors in some societies (24, 28). Analyzing data from two Japanese representative surveys that are the Comprehensive Survey of Living Conditions (CSLC) and the National Health and Nutritional Survey (NHNS), Fukuda and Hiyoshi noticed sex difference in the association between marital status and cardiovascular risk factors. They found that being married was associated with lower prevalence of cardiovascular risk factors among men and with higher prevalence of obesity, hypertension, and multiple risk factors among women (28). All these contradictions attest that the influence of marital status on arterial hypertension and cardiovascular risk factors need to be more specifically explored and explained.

Regarding family history of hypertension and body mass index, the results of the study showed that participants who reported a family history of hypertension were two times (*p* < 0.01) more susceptible to be hypertensive than those who did not. Similarly, overweight participants were 1.52 (p = 0.02) times more at risk of hypertension than those whose body mass indexes were equal to 25 kg/m^2^ or lower. The association of the two variables with hypertension is consistent with results of Wang et al. (2), Peer et al. (20), and Hammami et al. (25), suggesting that, there may be a single or combined effect of genetic and lifestyle factors in the genesis and the progression of high blood pressure.

The prevalence of hypertension was significantly higher in subjects with personal history of stroke than in those without (81.25 vs. 46.35%; *p* = 0.01). Although, this association appeared only in univariate analysis in the present study, hypertension is known to be a major risk factor for stroke (20, 25, 30). A study carried out in the United States showed that 77% of first-time stroke victims had high blood pressure (30). Another study carried out in South Africa indicated that the risk of being hypertensive was five times higher for patients suffering from stroke compared to others (31). Presented results are in line with these observations.

### Hypertension Awareness and Associated Factors

The importance of hypertension awareness toward its treatment and control is unquestionable, as awareness is the first stage of any disease management process. In resources-constrain settings, factors like misperceptions and lack of sensitization tend to alter people level of awareness (23, 31). Usually in the study area, people with high blood pressure do not express the need for management until complications occur. In this context, even blood pressure checkup for early high blood pressure detection is not frequent; people and stakeholders seeming disarrayed by infectious diseases. This could justify the low proportion of participants aware of their hypertensive status in the present study. The 36.85% proportion of participants aware of their status is lower than those reported in most of western countries (24, 32, 33). This difference could be explained by numerous economic and social facilities used to enhance sensitization, to carry out screenings, and to manage high blood pressure in western countries (1, 34, 35).

### Hypertension Treatment and Control

For better control of blood pressure, awareness is never sufficient if not followed by good therapeutic approach and follow-up. In our context, the management of hypertensive patients still a major challenge for caregivers as well as for the whole population. Presented results showed that 23.78% of aware hypertensive participants were using blood pressure lowering drugs. This fraction is close to the 30.5% reported in the VITARAA study (23), but it remains low as compared to that of western countries. Several factors may explain the low fraction of participants on antihypertensive treatment. Studies in low-income countries have shown that the high cost of drugs may be associated with the lack of treatment in known hypertensive patients (36–38). In our environment, low-cost antihypertensive (generic) drugs supply is not constant in public health facilities and some hypertensive patients are not able to buy their medicines all the times compromising therefore the better control of their blood pressure. Other factors such as lack of health insurance coverage for almost the entire population, local beliefs could explain low treatment rate observed (35, 36, 39).

For the treatment of hypertension, thiazide-type diuretics and angiotensin-converting enzyme inhibitors were the most frequently used antihypertensive agents in the study. The first group were used by 41.03% of the treated hypertensive participants and the second by 33.33%. These results are consistent with updated hypertension management recommendations (13, 40). This could be explained by the availability of some of these antihypertensive agents in generic form and the prescribing habits of general practitioners in our setting. Although calcium channel blockers have been shown to be one of the most effective antihypertensive agents in general black population (13), this group of drugs was not the most frequently used. In our context, nifedipine (10 mg short-acting nifedipine) still the common calcium channel blocker available in public health care facilities. Its principal mechanisms of action are peripheral and coronary vasodilation (41). Its harmful clinical effects include proischemic effect, complex ventricular tachyarrhythmias, Prohemorrhagic Effects, and marked hypotension (41). These side effects probably limit its current use for long term treatment of hypertension. Angiotensin receptor blockers were the least used (5.13%, *n* = 2), probably because of the high cost of this group of drugs.

The 28.21% hypertension control rate reported among treated hypertensive participants in the study was close to that of 24.6% reported by Dzudie et al. (3), but higher than those of 9.4% and 13.6% reported by Musinguzi and Nuwaha (6) and Katchunga et al. (23), respectively. The last two studies participants were recruited both in rural and urban area. In resource-constrain countries, rural areas are usually characterized by scarcity and remoteness of health facilities, very low resources, and poor level of education of the population. These factors may limit hypertension management and control thus, justifying the observed differences. The low hypertension control rate observed in the town of Ngaoundere may be due to the fact that people habitually put in competition traditional and modern medicine approaches of disease management. Nevertheless, the health facilities/population ratio is still low in this community and, there is a need to improve both medical staff training and health facilities technical platform for a better prevention and management of cardiovascular diseases.

### Strengths and Limitations

The present study is the first to analyze at one time data on the prevalence, awareness, and treatment of hypertension in a community setting in Ngaoundere. However, some limitations should be mentioned: the prevalence of hypertension was based on single blood pressure measurement and, treatment of hypertension was restricted to the use of antihypertensive agents, without consideration of non-pharmaceutical therapeutic strategies such as lifestyle modifications. Dietary factors and physical activity are important in pathogenesis and prevention of hypertension, but these were not assessed in the study. Moreover, it is known that economic factors play a significant role in access to health care especially in poor countries. Economic status of participants were not assessed in this study. In consequence, it was not possible to determine whether the participants who were aware and were on treatment had a better economic status or not.

## Conclusion

The present study revealed a high prevalence of hypertension and very low awareness, treatment, and control rates of this cardiovascular disease risk factor in Ngaoundere urban community. Further research is needed to identify various other factors associated with hypertension prevalence, awareness and control in resource – constrained African societies.

## Data Availability

All datasets analyzed for this study are included in the manuscript.

## Ethics Statement

Local Ethics Committee for Human Health Research, Reference no. 1121/L/RC/RA/DSP/HR/NGD/CLE. All the participants freely consented to participate in the study, after information and explanation given to them by the investigator.

## Author Contributions

OM contributed to the conception and study design, data interpretation, drafting of the manuscript, and approval of the final manuscript. TN contributed to the data collection and interpretation, drafting of the manuscript, and approval of the final manuscript.

### Conflict of Interest Statement

The authors declare that the research was conducted in the absence of any commercial or financial relationships that could be construed as a potential conflict of interest.
